# CircSMARCA5 Regulates VEGFA mRNA Splicing and Angiogenesis in Glioblastoma Multiforme Through the Binding of SRSF1

**DOI:** 10.3390/cancers11020194

**Published:** 2019-02-07

**Authors:** Davide Barbagallo, Angela Caponnetto, Duilia Brex, Federica Mirabella, Cristina Barbagallo, Giovanni Lauretta, Antonio Morrone, Francesco Certo, Giuseppe Broggi, Rosario Caltabiano, Giuseppe M. Barbagallo, Vittoria Spina-Purrello, Marco Ragusa, Cinzia Di Pietro, Thomas B. Hansen, Michele Purrello

**Affiliations:** 1 Department of Biomedical and Biotechnological Sciences—Section of Biology and Genetics “Giovanni Sichel”, University of Catania, 95123 Catania, Italy; dbarbaga@unict.it (D.B.); caponnettoangela@gmail.com (A.C.); duiliabrex@gmail.com (D.B.); mirabella.federica.91@gmail.com (F.M.); barbagallocristina@gmail.com (C.B.); giovannilau91@hotmail.it (G.L.); mragusa@unict.it (M.R.); dipietro@unict.it (C.D.P.); 2 Multidisciplinary Research Center on Brain Tumors Diagnosis and Therapy, University of Catania, 95123 Catania, Italy; cicciocerto@yahoo.it (F.C.); gbarbagallo@unict.it (G.M.B.); 3 Department of Medical, Surgical and Advanced Technological Sciences “G.F. Ingrassia”, University of Catania, 95123 Catania, Italy; morant592@libero.it (A.M.); giuseppe.broggi@gmail.com (G.B.); rosario.caltabiano@unict.it (R.C.); 4 Department of Biomedical and Biotechnological Sciences—Section of Medical Biochemistry, University of Catania, 95123 Catania, Italy; spinavit@unict.it; 5 Oasi Research Institute-IRCCS, 94018 Troina, Italy; 6 Department of Molecular Biology and Genetics (MBG), Aarhus University, 8000 Aarhus C, Denmark; 7 Interdisciplinary Nanoscience Center (iNANO), Aarhus University, 8000 Aarhus C, Denmark

**Keywords:** circular RNA, hsa_circ_0001445, RNA binding proteins, alternative splicing, glioblastoma multiforme, angiogenesis, VEGFA

## Abstract

Circular RNAs are a large group of RNAs whose cellular functions are still being investigated. We recently proposed that circSMARCA5 acts as sponge for the splicing factor Serine and Arginine Rich Splicing Factor 1 (SRSF1) in glioblastoma multiforme (GBM). After demonstrating by RNA immunoprecipitation a physical interaction between SRFS1 and circSMARCA5, we assayed by real-time PCR in a cohort of 31 GBM biopsies and 20 unaffected brain parenchyma controls (UC) the expression of total, pro-angiogenic (Iso8a) and anti-angiogenic (Iso8b) mRNA isoforms of Vascular Endothelial Growth Factor A (VEGFA), a known splicing target of SRSF1. The Iso8a to Iso8b ratio: (i) increased in GBM biopsies with respect to UC (*p*-value < 0.00001); (ii) negatively correlated with the expression of circSMARCA5 (*r*-value = −0.46, *p*-value = 0.006); (iii) decreased in U87-MG overexpressing circSMARCA5 with respect to negative control (*p*-value = 0.0055). Blood vascular microvessel density, estimated within the same biopsies, negatively correlated with the expression of circSMARCA5 (*r*-value = −0.59, *p*-value = 0.00001), while positively correlated with that of SRSF1 (*r*-value = 0.38, *p*-value = 0.00663) and the Iso8a to Iso8b ratio (*r*-value = 0.41, *p*-value = 0.0259). Kaplan-Meier survival analysis showed that GBM patients with low circSMARCA5 expression had lower overall and progression free survival rates than those with higher circSMARCA5 expression (*p*-values = 0.033, 0.012, respectively). Our data convincingly suggest that circSMARCA5 is an upstream regulator of pro- to anti-angiogenic VEGFA isoforms ratio within GBM cells and a highly promising GBM prognostic and prospective anti-angiogenic molecule.

## 1. Introduction

Circular RNAs (circRNAs) are a recently discovered large group of RNAs, of which biogenesis and functions within cells remain mostly to be investigated [1,2,3,4]. Notwithstanding the limited number of studies describing the mechanisms through which they act, the expression of several circRNAs has been found deregulated in pathological phenotypes from cancer to degenerative conditions: this suggests an active role of these molecules in their pathogenesis [5,6,7,8,9,10]. Splicing is thought to represent the main mechanism by which circRNAs originate [11,12,13]. Glioblastoma multiforme (GBM) is the most deadly human brain cancer to date: its median overall survival is 14.6 months after diagnosis and the prognosis is dismal in the majority of cases [14]. Notwithstanding the published data on the involvement of circRNAs and splicing in GBM, many questions remain without an answer [15,16,17,18]. Recently, we hypothesized that circSMARCA5 may carry out its function by tethering the splicing factor Serine and Arginine Rich Splicing Factor 1 (SRSF1) and that through this mechanism it may regulate GBM cells migration [19]. In addition to splicing, SRSF1 is involved in a plethora of other cellular functions as: (i) regulation of RNA metabolism; (ii) mRNA translation; (iii) miRNA processing; (iv) protein sumoylation; (v) stress response [20]. SRSF1 is also upregulated and acts as oncoprotein in several cancers [21]. Here, we demonstrate the physical interaction between circSMARCA5 and the splicing factor SRSF1 in GBM cells; based on our data, we propose that this interaction regulates the switch between pro- and anti-angiogenic isoforms generated by the splicing of the clinically relevant Vascular Endothelial Growth Factor A (VEGFA) pre-mRNA in GBM cells.

## 2. Results

### 2.1. SRSF1 and CircSMARCA5 Physically Interact within GBM Cells

**Figure 1:**
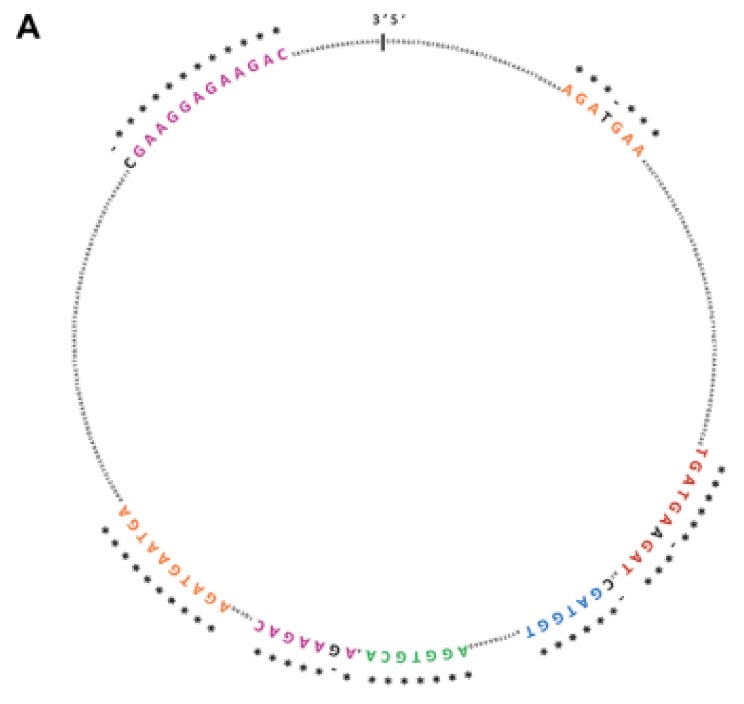

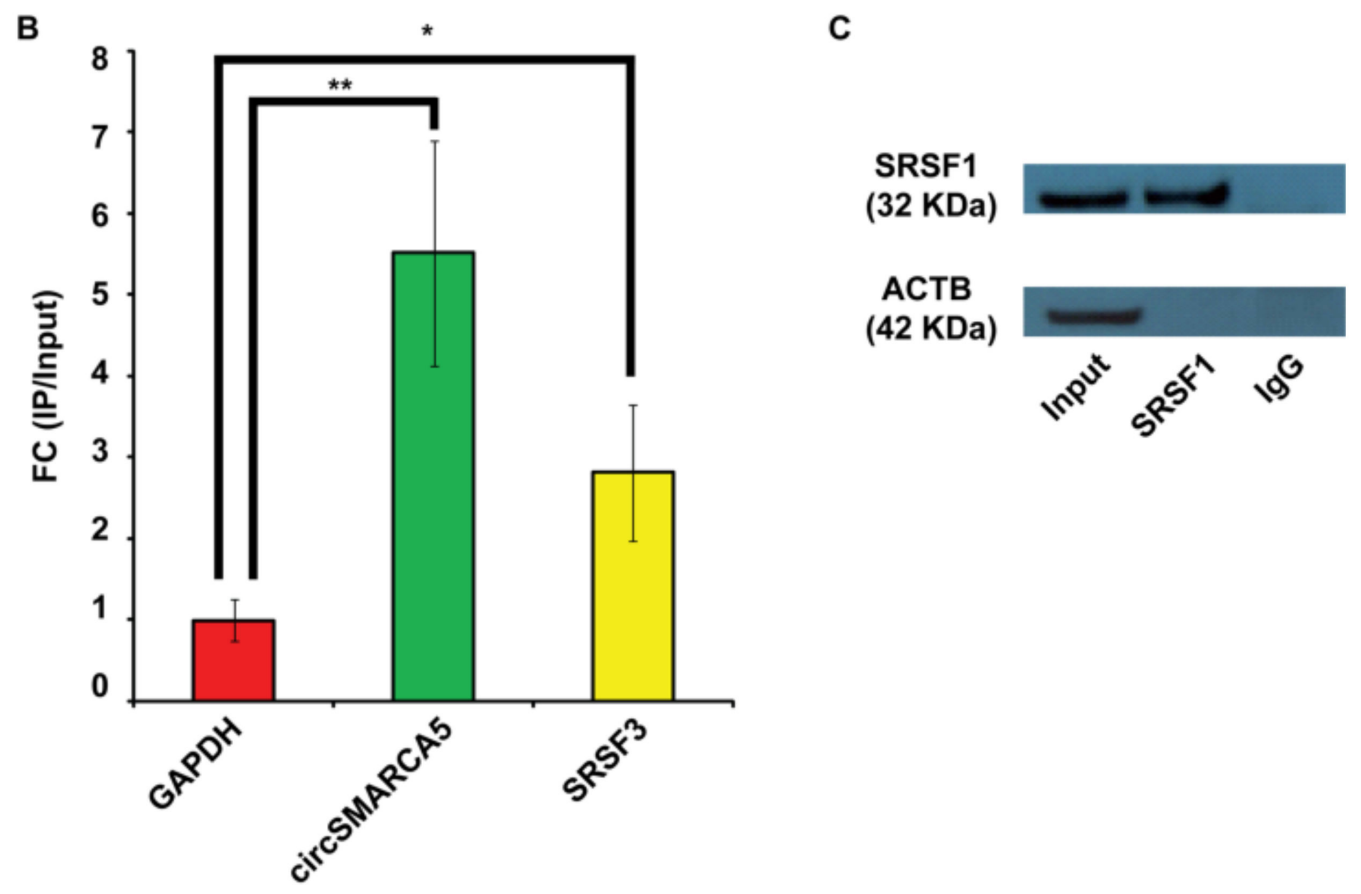
CircSMARCA5 and SRSF1 physically interact within U87-MG cells. (**A**) SRSF1 binding sites predicted by RBPMap database (http://rbpmap.technion.ac.il/) are highlighted with different colors within circSMARCA5 sequence. * indicates sequence conservation among twelve primates, including *Homo sapiens* (see [19]). (**B**) Fold enrichment (FC) of circSMARCA5, SRSF3 and GAPDH are shown as IPed samples/input. See [22] and Section 4 Materials and Methods for further details (* *p*-value < 0.05; ** *p*-value < 0.01, *N* = 4, two-samples *t*-test). (**C**) Representative western blot of U87-MG Input, SRSF1 and normal IgG RIPed samples. Antibodies against SRSF1 and Actin Beta (ACTB) were used as described in Section 4 Materials and Methods.

CircSMARCA5 was predicted to be bound by SRSF1 in at least seven different evolutionarily conserved sites (see [19] and Figure 1A). SRSF1 RNA immunoprecipitation (RIP) allowed us to validate this interaction, showing a significant enrichment of circSMARCA5 in addition to SRSF3 mRNA, a known splicing target of SRSF1 (positive control), in immunoprecipitated U87-MG cells lysate with respect to a negative control (Figure 1B,C).

### 2.2. SRSF1 and VEGFA Are Upregulated While circSMARCA5 Is Downregulated in GBM Biopsies

**Figure 2:**
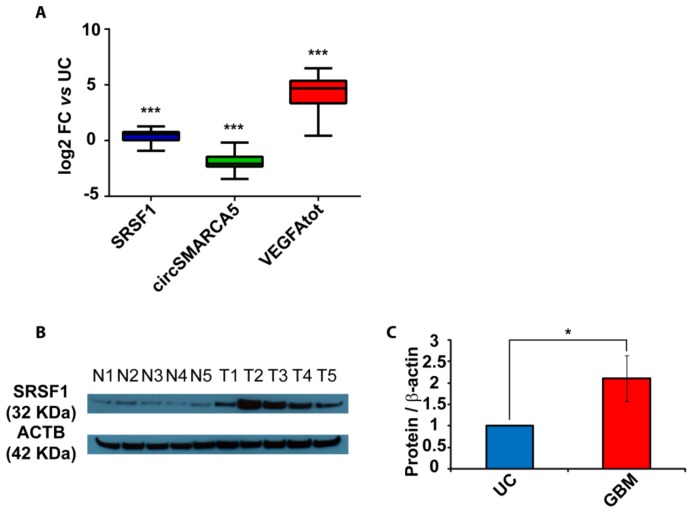
SRSF1, circSMARCA5 and VEGFA expression in GBM biopsies and unaffected controls (UC). (**A**) Box-and-whisker plots, representing the expression of SRSF1, circSMARCA5 and total VEGFA RNA in the studied cohort. Data are represented as log_2_ fold change (FC) values versus UC. (*** *p*-value < 0.001, N_(GBM)_ = 31, N_(UC)_ = 20, Mann–Whitney test). (**B**) Western blot of SRSF1 in a selection of UC (N) and GBM (T) samples. ACTB was used as a loading control. (**C**) Bar graph representing densitometric quantification of SRSF1. Data, shown as mean ± standard deviation, represent fold change versus UC (* *p*-value < 0.05, N_(GBM)_ = 14, N_(UC)_ = 8, two-sample *t*-test).

SRSF1 was upregulated in GBM biopsies with respect to unaffected brain parenchyma (UC), both as mRNA (*p*-value = 0.0009, Mann–Whitney test) and protein (*p*-value = 0.022, two-sample *t*-test) (Figure 2A–C). After a literature review of SRSF1’s splicing targets (Table S1, Supplementary Materials), we focused on VEGFA, a molecule of high clinical interest in GBM (see Discussion). We demonstrated that total VEGFA mRNA was upregulated in the same cohort (*p*-value < 0.00001, Mann–Whitney test), while circSMARCA5 was downregulated (*p*-value < 0.00001, Mann–Whitney test), confirming our data previously obtained in an independent GBM cohort (Figure 2A). Upregulation of SRSF1 and VEGFA mRNA, as well as their positive correlation, was also observed in the extended cohort of GBM biopsies analyzed in REpository for Molecular BRAin Neoplasia DaTa (REMBRANDT) (Figure S1, Supplementary Materials) and The Cancer Genome Atlas (TCGA) (Figure S2, Supplementary Materials) databases. Most specifically, based on REMBRANDT data, SRSF1 mRNA was significantly upregulated in all glioma grades with respect to normal brain (*p*-value < 0.0001, ANOVA test) (Figure S1A, Supplementary Materials), while VEGFA was significantly upregulated specifically in GBM (*p*-value < 0.0001, ANOVA test) (Figure S1B, Supplementary Materials). In more detail, SRSF1 and VEGFA mRNAs were both significantly upregulated in all GBM subtypes, except for the neural subtype, with respect to unaffected brain samples (see significant *p*-values within Figure S2, Supplementary Materials) [23,24].

### 2.3. The Ratio of Pro- to Anti-Angiogenic VEGFA mRNA Isoforms Increases in GBM Cells as Compared to Unaffected Brain Parenchyma and Decreases in U87-MG Overexpressing circSMARCA5

**Figure 3:**
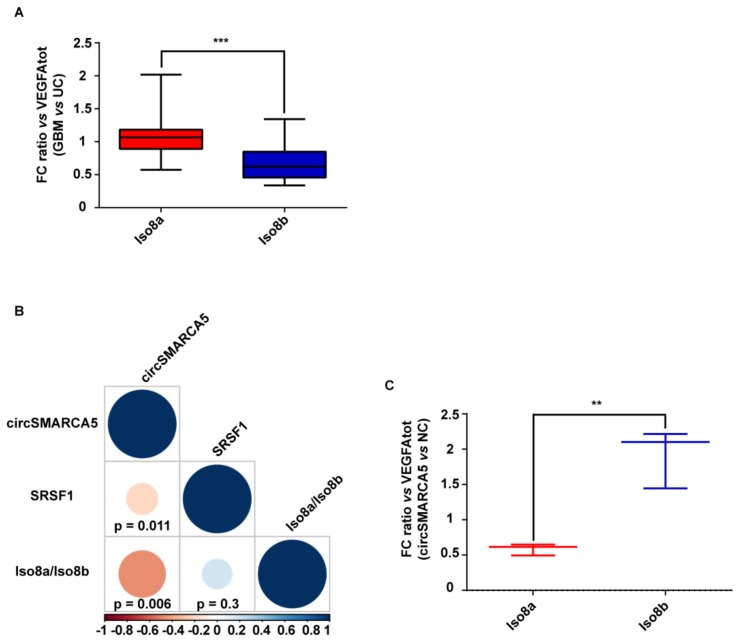
Iso8a-to-Iso8b ratio in GBM biopsies vs. UC. (**A**) Box-and-whisker plots, representing the ratios between fold changes of Iso8a and total VEGFA (VEGFA_tot_) and Iso8b and VEGFA_tot_ in GBM compared to UC (*** *p*-value < 0.00001, *N* = 27, Mann–Whitney test) (see Section 4 Materials and Methods for details on data analysis). (**B**) Correlation matrix among the expression of circSMARCA5 and SRSF1 and Iso8a-to-Iso8b ratio. Positive and negative correlations are displayed in blue and red colors, respectively. The color scale bar indicates r values. Color intensity and the size of the circle are proportional to the correlation coefficients into the correlogram. (**C**) Box-and-whisker plots, representing the ratios between fold changes of Iso8a and VEGFA_tot_ and Iso8b and VEGFA_tot_ in U87-MG overexpressing circSMARCA5 with respect to U87-MG transfected with the empty vector (NC) (** *p*-value < 0.001, *N* = 3, two-sample *t*-test) (see Section 4 Materials and Methods for details on data analysis).

In order to define the role of circSMARCA5 in the control of VEGFA mRNA splicing, the expression of total VEGFA, pro- and anti-angiogenic VEGFA mRNA isoforms (Iso8a and Iso8b, respectively) were assayed in the same cohort previously analyzed. Iso8a isoforms were most expressed in all biopsies, as shown by real-time PCR data. Most specifically, the Iso8a-to-Iso8b ratio was significantly higher in GBM biopsies with respect to UC (*p*-value < 0.00001, Mann–Whitney test) (Figure 3A and Figure S3) (see Section 4 Materials and Methods for details on data analysis). In the same cohort, the expression of circSMARCA5 negatively correlated with that of SRSF1 mRNA (*r*-value = −0.36, *p*-value = 0.011, Spearman correlation test) and with the Iso8a-to-Iso8b ratio (*r*-value = −0.47, *p*-value = 0.006, Spearman correlation test) (Figure 3B). The Iso8a-to-Iso8b ratio was also higher in three GBM cell lines (A172, CAS-1 and U87MG) with respect to unaffected brain (Figure S4, Supplementary Materials). Interestingly, U87-MG overexpressing circSMARCA5 showed a significant decrease in the Iso8a-to-Iso8b ratio with respect to the same cells transfected with the empty vector (negative control, NC) (*p*-value = 0.0055, two-sample *t*-test) (Figure 3C; Figures S3 and S5, Supplementary Materials).

### 2.4. Blood Vascular Microvessel Density (MVD) Negatively Correlates with the Expression of circSMARCA5, While Positively Correlating with SRSF1 mRNA Expression and Pro/Anti-Angiogenic VEGFA Isoforms Ratio

**Figure 4:**
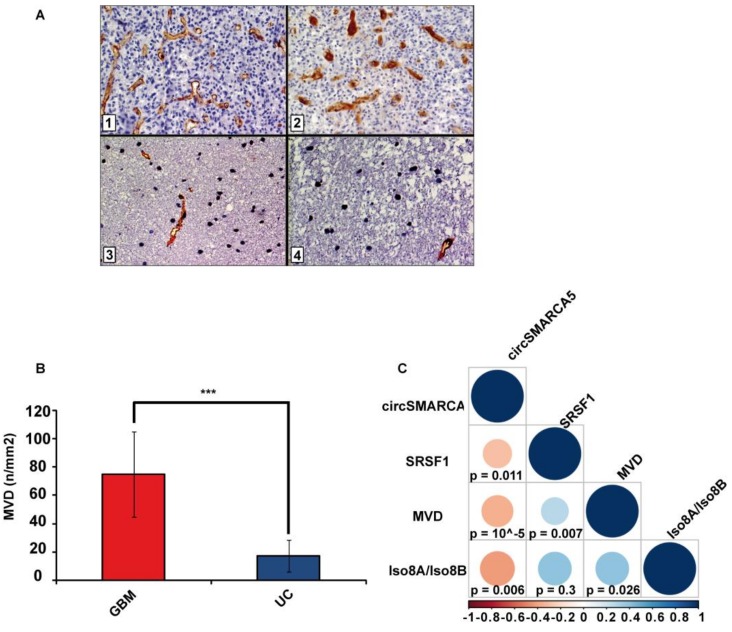
MVD in GBM and UC samples. (**A**) Representative immunohistochemical staining for CD31 showing areas of high MVD with multiple branching vessels in glioblastoma tissue (1 and 2) and a lower MVD in unaffected brain tissue (3 and 4). Immunoperoxidase staining; 400× magnification (**B**) Bar graph representing the mean MVD in GBM and UC samples. Data are represented as mean ± standard deviation (*** *p*-value < 0.00001, N_(GBM)_ = 31, N_(UC)_ = 18, two sample *t*-test). (**C**) Correlation matrix among MVD, circSMARCA5 and SRSF1 expression and Iso8a to Iso8b ratio. Positive and negative correlations are displayed in blue and red color, respectively. Color scale bar indicates r values. Color intensity and the size of the circle are proportional to the correlation coefficients into the correlogram.

As expected, MVD evaluated in the same cohort previously characterized was significantly higher in GBM samples than in UC (*p*-value < 0.00001, two-sample *t*-test) (Figure 4A,B). MVD positively correlated with the SRSF1 mRNA expression and the Iso8a-to-Iso8b ratio (*r*-values = 0.38, and 0.41, respectively; *p*-values = 0.00663, and 0.026, respectively, Spearman correlation test), while negatively correlating with circSMARCA5 expression (*r*-value = −0.59, *p*-value = 0.00001, Spearman correlation test) (Figure 4C).

### 2.5. CircSMARCA5 Expression Negatively Correlates with GBM Patients’ Overall Survival (OS) and Progression-Free Survival (PFS)

**Figure 5:**
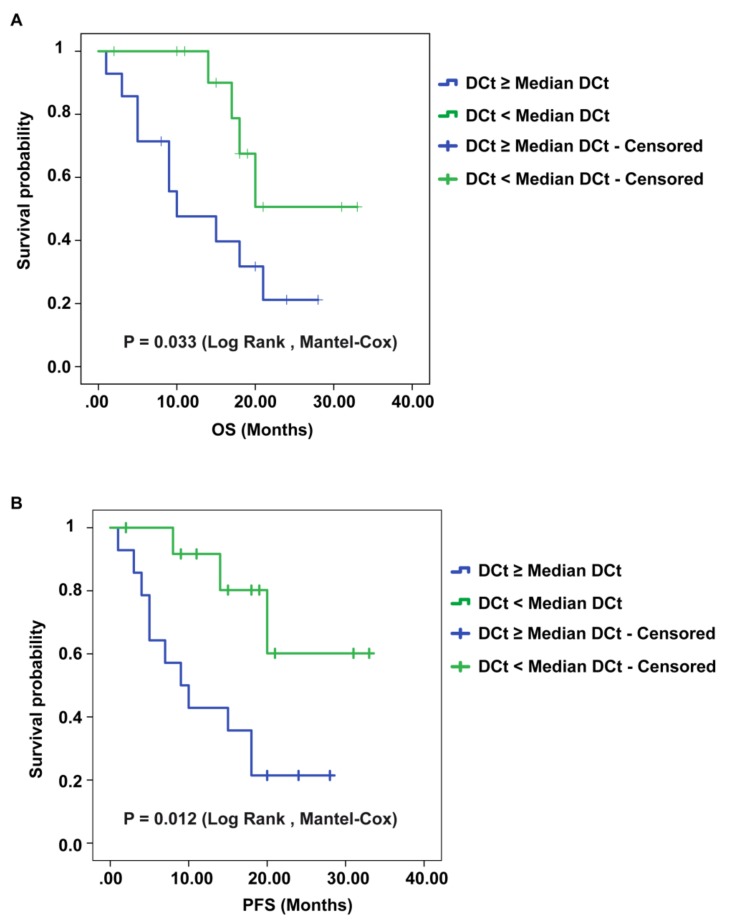
Kaplan–Meier overall survival (OS) and progression-free survival (PFS) curves of GBM patients, based on the expression of circSMARCA5. Patients having a lower expression of circSMARCA5 (DCt ≥ Median DCt) survive less (**A**) show a shorter PFS (**B**) than patients with a higher expression of circSMARCA5 (DCt < Median DCt).

We found that lower circSMARCA5 expression was associated with poorer OS and PFS in GBM (*p*-values = 0.033, and 0.012, respectively, logrank test) (Figure 5A,B). Kaplan–Meier analysis performed on TCGA data showed also a negative correlation between mesenchymal GBM patients’ OS and SRSF1 expression (*p*-value = 0.0172, logrank test) (Figure S6, Supplementary Materials).

## 3. Discussion

As previously described by our group, circSMARCA5 sequence is predicted to be enriched in SRSF1 protein-binding sites: accordingly, a physical interaction between these two molecules has been identified by high-throughput enhanced UV crosslinking and immunoprecipitation (eCLIP) analysis in K562 cells [19,25]. Deeper knowledge on the interactions between RNAs and RNA-binding proteins (RBPs) in specific cell contexts is a critical piece of the cell function puzzle [26]. For instance, the ability of specific viral RNAs to function as a sponge for several host cell’s RBPs has been extensively analyzed [27]; similarly, many coding and non-coding RNAs have been described to behave as miRNAs- and RBPs-sponges in eukaryotic cells [28,29]. Here, we validate, through RIP data, the physical interaction between circSMARCA5 and SRSF1 in human GBM cells. SRSF1 is a protein involved in many biomolecular functions [20] and its expression is known to be upregulated in several cancers [30,31]. In order to understand if the interaction between circSMARCA5 and SRSF1 may alter the splicing pattern of SRSF1’s targets, we focused on VEGFA. It is known that VEGFA pre-mRNA can be alternatively spliced generating both pro-angiogenic isoforms (VEGF-A_xxx_a) and anti-angiogenic isoforms (VEGF-A_xxx_b), depending on the recognition of a proximal splicing site (PSS) within the eighth exon of VEGFA pre-mRNA by SRSF1: specifically, the higher the amount of SRSF1 binding this PSS, the higher the retention of full-length eighth exon is, improving the synthesis of VEGF-A_xxx_a isoforms [32,33,34]. Aberrant splicing of VEGFA, leading to an alteration in the pro/anti-angiogenic ratio was described in human colon cancer [35]. Data from biopsies and our in vitro model following overexpression of circSMARCA5, together with our previous data [19], support the hypothesis that this circRNA performs a *trans*-acting splicing function within GBM cells, which it carries out by tethering SRSF1. Moreover, this pathway seems to be specifically deregulated in GBM cells, among the other glioma grades, based on our previous observations and on REMBRANDT and TCGA data. Additionally, the hypothesis of a functional involvement of circSMARCA5 in the regulation of angiogenesis is supported by correlations observed among the circSMARCA5/SRSF1/VEGFA expression and the blood microvascular vessel density. This is an important aspect of GBM biology: ultimately, the control of the pro- to anti-angiogenic switch may represent a valid alternative to the therapy based on monoclonal anti-VEGFA antibodies that did not reach the expected results to date [36]. Finally, even if on a limited cohort of patients, the perspective use of circSMARCA5 as a prognostic biomarker and a therapeutic target [33,37,38] in GBM cells appears to be promising.

## 4. Materials and Methods

### 4.1. GBM Biopsies

Each specimen obtained during intraoperative navigation-assisted neurosurgery was divided in two halves: one half was supplied to pathologists for fixation and tissue embedded into paraffin, and the other was immediately frozen and stored at −80 °C until use. All patients enrolled in this study supplied their written informed consent before surgery. The study was conducted in accordance with the declaration of Helsinki and the protocol was approved by the ethical committee of Azienda Ospedaliero-Universitaria "Policlinico-Vittorio Emanuele", Catania, Italy. Only biopsies from patients with a confirmed pathological diagnosis of GBM were included into the study (*N* = 31). Unaffected brain parenchyma was obtained, when possible, from a non-eloquent region of the brain, adjacent to the tumor and negative to 5-aminolevulinic acid (5-ALA) fluorescence: this type of sample has been defined as unaffected control and used as calibrator tissue in this study only after pathologists observed no infiltration of cancer cells (*N* = 20). Clinical data from patients enrolled in the study are summarized in Table 1.

### 4.2. Cell Cultures and Transfection

GBM cell lines A172, CAS-1 and U87-MG culture and transfection with pcDNA3-circSMARCA5 or empty pcDNA3 vectors were performed as previously described [10,19].

### 4.3. RNA Immunoprecipitation (RIP)

Briefly, cells were seeded in 10 cm dishes at a density of 3.6 × 10^6^ and cultured for 72 hours. RIP was performed as previously described by Peritz et al. [39], with some modifications. More specifically, RIP was performed without cross-linking. Immunoprecipitation was performed using 5 μg of mouse monoclonal IgG2b antibody against SRSF1 (Santa Cruz Biotechnology, Inc., Heidelberg, Germany, Cat. n. sc-73026) or isotype control IgG from mouse (negative control) (Santa Cruz Biotechnology, Inc., Cat. n. sc-2025). Data were analyzed as described by Ratnadiwakara et al. [22]. RIP methodology and data analysis are fully described in Supplementary Materials.

### 4.4. RNA Extraction and Real-Time PCR

RNA was extracted by using Trizol (ThermoFisher Scientific, Waltham, MA USA), according to manufacturer’s instruction and quantified both by spectrophotometer and Qubit™ fluorometer (ThermoFisher Scientific). A commercially available RNA from human brain (Ambion, Austin, TX, USA) has been used as further unaffected control. Real-time PCR was performed as previously described [40] and relative RNA amounts were estimated by using 2^-DDCt^ method [41]. For a fully description of real-time PCR data analysis within this manuscript, see Supplementary Materials. Linear and circular RNAs were amplified by using convergent and divergent primers, respectively, as described in Table S2 and in Figure S7 (Supplementary Materials).

### 4.5. Protein Extraction and Immunoblotting

Proteins from biopsies were extracted by using RIPA buffer (Abcam, Cambridge, UK) and quantified by Qubit™ fluorometer (ThermoFisher Scientific). Human Brain Cerebral Cortex Protein Medley (Takara Clontech^®^, Mountain View, CA, USA) was used as further unaffected control. Western blot analysis was performed as previously described [42]. Primary antibodies against the following proteins were used: SRSF1 (mouse monoclonal antibody from Santa Cruz Biotechnology, Inc., Cat. n. sc-73026) and ACTB (rabbit polyclonal antibody from Abcam, Cat. n. ab16039). Secondary antibodies were HRP-conjugated anti-mouse (for SRSF1) or anti-rabbit (for ACTB) (Santa Cruz Biotechnology, Inc, Cat. n. sc-516102 and sc-2004, respectively) for chemiluminescent detection. Gel bands were quantified by ImageJ software (https://imagej.nih.gov/ij/index.html).

### 4.6. Immunohistochemistry

For each case, all Hematoxylin and Eosin (H&E) stained sections were assessed by two pathologists and one representative sample was identified. Sections were processed as previously described [43]. Briefly, slides were cut at 4–5 µm, dried, deparaffinized and rehydrated. Then, sections were incubated for 30 min at 4 °C with mouse monoclonal anti-Human CD31, Endothelial Cell antibody (JC70A, Dako Corporation, Glostrup, Denmark), diluted 1/40 in PBS (Sigma, Milan, Italy). The biotinylated anti-rabbit secondary antibody was applied for 30 min at 20 °C, followed by the avidin–biotin–peroxidase complex (Vector Laboratories, Burlingame, CA, USA) for a further 30 min at 20 °C. The immunoreaction was visualized by incubating the sections for 4 min in a diaminobenzidine (DAB) and 0.02% hydrogen peroxide solution (DAB substrate kit, Vector Laboratories, CA, USA).

### 4.7. Assessment of Blood Vascular Microvessel Density (MVD)

MVD of CD31 was evaluated by two pathologists. Immunohistochemical sections were observed with a Zeiss Axioplan light microscope (CarlZeiss, Oberkochen, Germany). MVD assessment was performed as described by Weidner et al. and Mikkelsen et al., with some modifications [44,45]. Briefly, vascular hotspots were identified on CD31 sections by a light microscope at 4× and 10× magnifications. MVD was evaluated as the total number of vessels per mm^2^ with a conversion factor of 1 mm^2^ equaling 4 high power fields (HPFs) at 40× magnification of highest vascular densities [46]. Areas with ≥50 of viable tumor cells were counted; tissues with extensive necrosis, hemorrhage and desmoplasia were excluded. Every single stained endothelial cell and each lumen for long branched vessels and glomeruloid tufts were counted. Moreover, small clusters of ≥2 staining endothelial cells within the same vessel was counted as one vascular structure.

### 4.8. Statistics

All the statistical tests used in this study are described throughout the text and in figure legends. *p*-values lower than 0.05 were considered significant.

## 5. Conclusions

We proposed that our data convincingly suggest that circSMARCA5 is an upstream regulator of the ratio of the pro- and anti-angiogenic VEGFA isoforms within GBM cells and is a promising prognostic biomarker and a prospective therapeutic target in GBM cells.

## Figures and Tables

**Table 1:** Clinical data of Glioblastoma (GBM) and control samples.

Sample	N	Mean Age (Years ± Std. Dev.)	Sex		Mean OS (Months ± Std. Dev.)	Mean PFS (Months ± Std. Dev.)
			M	F		
Fresh frozen GBM biopsies	31	63.6 ± 10.9	15	16	15 ± 8.2	13.8 ± 8.7
Fresh frozen unaffected brain parenchyma	20	64 ± 10.3	8	12		
FirstChoice^®^ Human Brain Reference RNA	1 (commercially available)	68.3 ± 15	13	10		

## Supplementary Materials

The following are available online at http://www.mdpi.com/2072-6694/11/2/194/s1, Table S1, List of SRSF1’s splicing targets retrieved by literature. Table S2, Sequences of primers used in the study. Figure S1, SRSF1 and VEGFA mRNA expression in different types of glioma (REMBRANDT database). Figure S2, SRSF1 and VEGFA mRNA expression in GBM subtypes (TCGA database). Figure S3, Box-and-whisker plots, representing the Iso8a/Iso8b ratio in GBM and UC. Figure S4, Bar graph showing Iso8A vs Iso8B ratio in three different GBM cell lines. Figure S5, CircSMARCA5 but not linear SMARCA5 is overexpressed in U87-MG transfected with pcDNA3-circSMARCA5 vector with respect to NC (U87-MG transfected with the empty vector). Figure S6, Kaplan-Meier overall survival curves of mesenchymal GBM patients, based on the expression of SRSF1. Figure S7, Diagram of primers used to amplify Iso8a and Iso8b VEGFA specific isoforms by qRT PCR.
