# Supplementary material for: CircSMARCA5 Regulates VEGFA mRNA Splicing and Angiogenesis in Glioblastoma Multiforme Through the Binding of SRSF1
Source: Cancers (Basel). 2019 Feb 7;11(2):194. doi: 10.3390/cancers11020194 (PMC6406760; doi:10.3390/cancers11020194)
Supplement: Supplementary file 1 [file cancers-11-00194-s001.pdf]

cancers

MDPI

Supplementary Material

CircSMARCA5 Regulates VEGFA mRNA Splicing and Angiogenesis in Glioblastoma Multiforme Through the binding of SRSF1

Davide Barbagallo, Angela Caponnetto, Duilia Brex, Federica Mirabella, Cristina Barbagallo, Giovanni Lauretta, Antonio Morrone, Francesco Certo, Giuseppe Broggi, Rosario Caltabiano, Giuseppe M. Barbagallo, Vittoria Spina-Purrello, Marco Ragusa, Cinzia Di Pietro, Thomas B Hansen and Michele Purrello

Supplementary Methods:

RNA Immunoprecipitation (RIP)

Twenty microliters of Dynabeads™ Protein A/G (ThermoFischer Scientific) were firstly equilibrated with lysis buffer and then incubated with 5 µg of mouse monoclonal IgG2b antibody against SRSF1 (Santa Cruz Biotechnology, Inc., Heidelberg, Germany) or isotype control IgG from mouse (negative control) (Santa Cruz Biotechnology, Inc.) for 2 h at 4 °C. Ten percent of the volume of U87-MG lysate supernatant were collected before immunoprecipitation and used as Input for RNA and protein analyses, respectively. The remaining supernatant was divided in two aliquots that were incubated either with monoclonal IgG2b antibody against SRSF1 or isotype control IgG for 2 h at 4 °C. After washing, ten percent of the beads were used for western blot analysis of either SRSF1 or IgG pulled-down. The remaining beads were resuspended in 1 mL of Trizol for RNA extraction. Western blot analysis was performed on Input, SRSF1- and IgG-IPed samples in order to verify the specificity of immunoprecipitation (see Figure 1C). Real time PCR data analysis was performed as described by Ratnadiwakara M and Coll. [22]. More in details, DCt was calculated as $Ct_{Ip\ (either\ SRSF1\text{-}IPed\ or\ IgG\text{-}IPed)} - Ct_{Input}$ for each transcript assayed (GAPDH, SRSF3 and circSMARCA5). Then DDCt were calculated as $DCt_{SRSF1\text{-}IPed} - DCt_{IgG\text{-}IPed}$. Finally, fold change (FC) was calculated as 2^{-DDCt}. GAPDH FC was set to one and FC of circSMARCA5 and SRSF3 mRNA (a known interactant of SRSF1 protein, used as positive control) were calculated accordingly. Data were shown as average fold changes of four replicates (see Figure 1B).

Real-Time PCR Data Analysis of Total VEGFA, Iso8a and Iso8b Isoforms

Primers used to amplify $VEGFA_{tot}$ were designed on exons 2 (forward) and 3 (reverse), which are common to all the human VEGFA isoforms annotated in Gene NCBI database (https://www.ncbi.nlm.nih.gov/gene/7422). A common forward primer recognized both Iso8a and Iso8b isoforms, while specific reverse primers were used to amplify either Iso8a or Iso8b, as described in Figure S6. The Iso8a to Iso8b ratio in GBM vs UC or in U87-MG overexpressing circSMARCA5 vs NC was estimated through two different approaches: (i) based on the ratios between fold changes of both Iso8a and $VEGFA_{tot}$ and Iso8b and $VEGFA_{tot}$ in GBM (or U87-MG overexpressing circSMARCA5) vs UC (or NC) (see Figures 3A and 3C); (ii) based on direct comparison of the amount of Iso8a and Iso8b (Iso8a/Iso8b) in GBM, UC, U87-MG overexpressing circSMARCA5 and NC (see Figure S3). More in details, in the first approach fold change (FC) expression (calculated as 2^{-DDCt}) of $VEGFA_{tot}$, Iso8a and Iso8b were firstly evaluated in GBM (or U87-MG overexpressing circSMARCA5) vs UC (or NC, U87-MG transfected with the empty vector), then FC Iso8a/ FC $VEGFA_{tot}$ and FC Iso8b/ FC $VEGFA_{tot}$ ratios were calculated and shown in the graph (see Figures 3A,C). In the second approach, the ratio between 2^{-DCt} of Iso8a and 2^{-DCt} of Iso8b was calculated for each sample and reported in the graph as box-plot for each type of sample (GBM,

UC, U87-MG overexpressing circSMARCA5 and NC). TBP mRNA was used as endogenous control, in order to obtain DCts. Statistical analysis was performed as described in figure legends.

Table S1. List of SRSF1's splicing targets retrieved by literature.

SRSF1 target's Official Gene Symbol	Reference (PMID or DOI)
ADD1	17310252
AKT	26273603
AKT1	26431027
ANXA7	24550987
BCL2	28315432
BCL2A1	28315432
BCL2L1	26273603
BCL2L11	22245967
BCL2L2	28315432
BIN1	17310252
BIRC5	24550987
CASP2	24807918
CASP2	17310252
CASP8	28315432
CASP9	26273603
CCND1	23592547
CD247	24807918
CD44	24807918
CDK4	18841201
CDKN1A	25993413
CEBPA	28315432
CFLAR	28315432
CLK1	24842991
CRADD	28315432
CTNNB1	23592547
DFFA	26273603
DIABLO	28315432
EGFR	DOI: 10.18103/mra.v0i1.11
ENG	24807918
ENSA	18841201
FAS	28315432
FGFR	DOI: 10.18103/mra.v0i1.11
FN1	21615404
FOXO4	26431027
HIPK2	28315432
HNRNPA2B1	17310252
IGF1R	18841201
MADD	28315432
MAPK3	18841201
MAPT	24807918
MAX	DOI: 10.18103/mra.v0i1.11
MCL1	24550987
MIR505	29120871
MKNK2	17310252
MNK2B	26273603
MST1R	16364913
MYC	28315432
NETO2	18841201
PABPC1	18841201
PDCD4	28315432

PKM	24842991
PRKCD	26431027
PRKDC	28315432
RAC1	19602482
RPS6KB	17310252
RPS6KB1	25776557
RTN4	DOI: 10.18103/mra.v0i1.11
SFRS1	18841201
SLC39A14	24807918
SRSF3	9305649
TEAD1	26273603
TEAD1	17310252
TMPO	28315432
TNFRSF19	28315432
TNFRSF9	28315432
TNFSF10	28315432
TP53	28315432
TPM1	DOI: 10.18103/mra.v0i1.11
TSC2	17310252; 26431027
VEGFA	26273603

Table S2. Sequences of primers used in the study.

Transcript	Fw primer	Rev primer
linearSMARCA5	ATGGGTACCAACACTTAGATCTGT	AACGTCTCTGACAAAAGCAGC
circSMARCA5	ACAATGGATACAGAGTCAAGTGTT	CCACAAGCCTCCCTTTTGTTTT
GAPDH	GTCAGCCGCATCTTCTTTTG	GCGCCCAATACGACCAAATC
SRSF1	CCATCCAGGCGGTCTGAAAA	ACCTGCTTCACGCATGTGAT
SRSF3	TCGTCGCCCTCGAGATGAT	GTGGTGAGAAGAGACATGATGGT
VEGFA Iso8a	TTCCTGCAAAAACACAGACTCGC	TCACCGCCTCGGCTTGTCACAT
VEGFA Iso8b	TTCCTGCAAAAACACAGACTCGC	TCAGTCTTTCCTGGTGAGAGATCTGCA
VEGFAtot	GCACCCATGGCAGAAGG	CTCGATTGGATGGCAGTAGCT

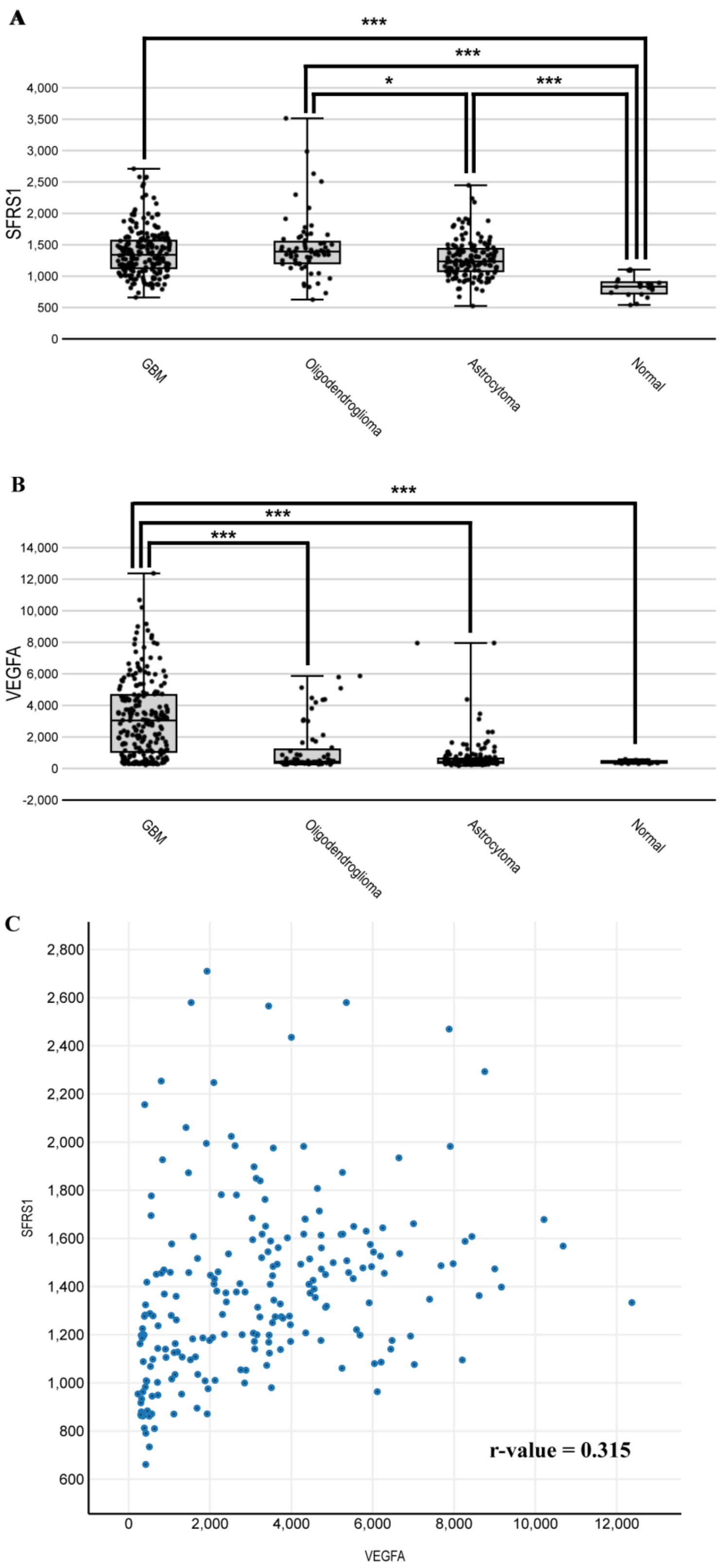

Figure S1. SRSF1 and VEGFA mRNA expression in different types of glioma (REMBRANDT database). (**A**) Box-and-whisker plots, representing the expression of SRSF1 (Affymetrix HG U133 v2.0 Plus) (* p-value < 0.05; *** p-value < 0.001, ANOVA Dunn's multiple comparisons test); (**B**) Box-and-whisker plots, representing the expression of VEGFA (Affymetrix HG U133 v2.0 Plus) (*** p-value < 0.001, ANOVA Dunn's multiple comparisons test); (**C**) Scatter plot representing the correlation between SRSF1 and VEGFA expression in GBM samples.

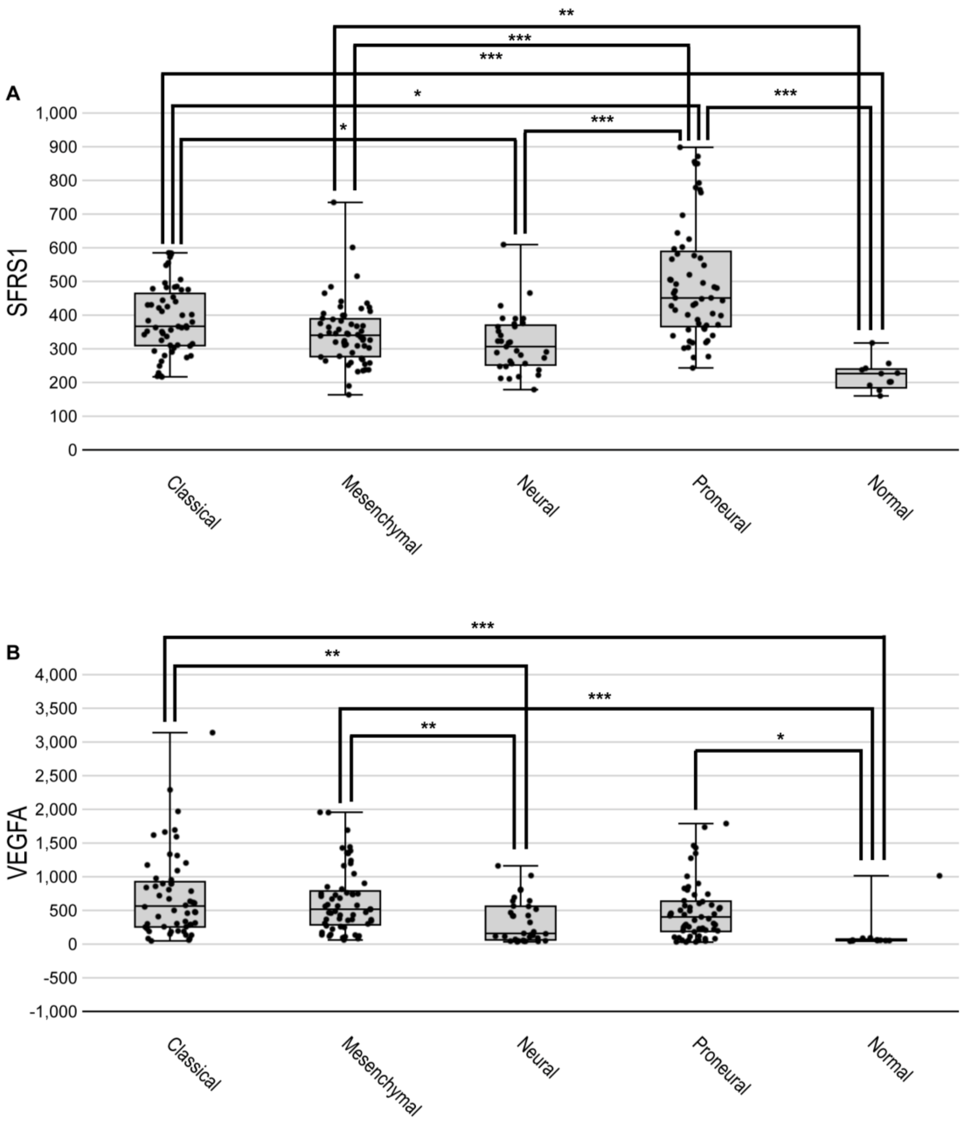

Figure S2. SRSF1 and VEGFA mRNA expression in GBM subtypes (TCGA database). (**A**) Box-and-whisker plots, representing the expression of SRSF1 (Affymetrix HT HG U133A) (* *p*-value < 0.05; ** *p*-value < 0.01; *** *p*-value < 0.001, ANOVA Dunn's multiple comparisons test); (**B**) Box-and-whisker plots, representing the expression of VEGFA (Affymetrix HT HG U133A) (* *p*-value < 0.05; ** *p*-value < 0.01; *** *p*-value < 0.001, ANOVA Dunn's multiple comparisons test).

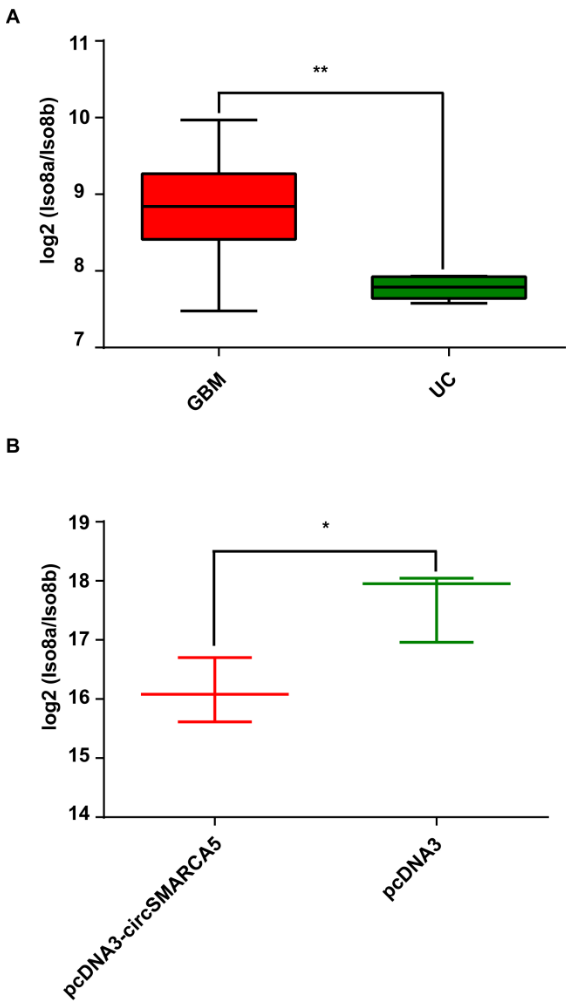

Figure S3. Box-and-whisker plots, representing the Iso8a/Iso8b ratio in GBM and UC (**A**) and in U87-MG overexpressing circSMARCA5 (pcDNA3-circSMARCA5) and NC (pcDNA3) (**B**). Data are shown as $\log_2 (2^{-DCt}(Iso8a)/2^{-DCt}(Iso8b))$. See Supplementary Methods for further details. (** p-value < 0.01, $N_{(GBM)} = 27$, $N_{(UC)} = 5$, Mann-Whitney test (**A**); (* p-value < 0.05, N = 3, two sample t-test (**B**)).

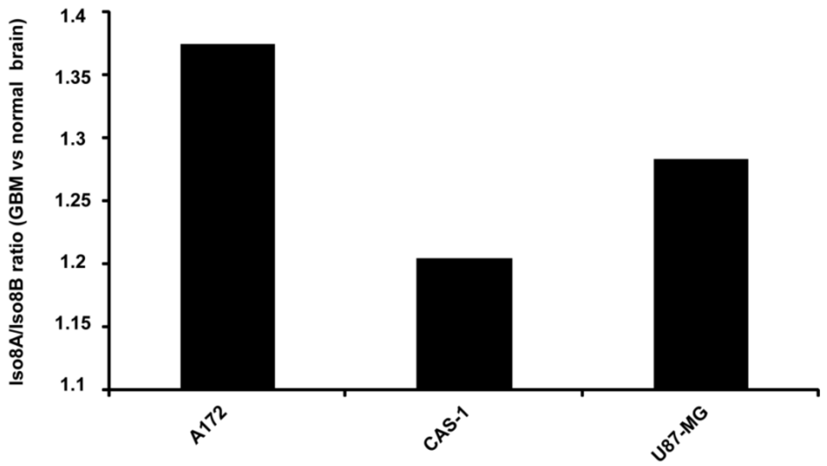

Figure S4. Bar graph showing Iso8a vs Iso8b ratio in three different GBM cell lines. Total VEGFA was used as endogenous control; normal brain from Ambion (see Materials and Methods) was used as calibrator tissue.

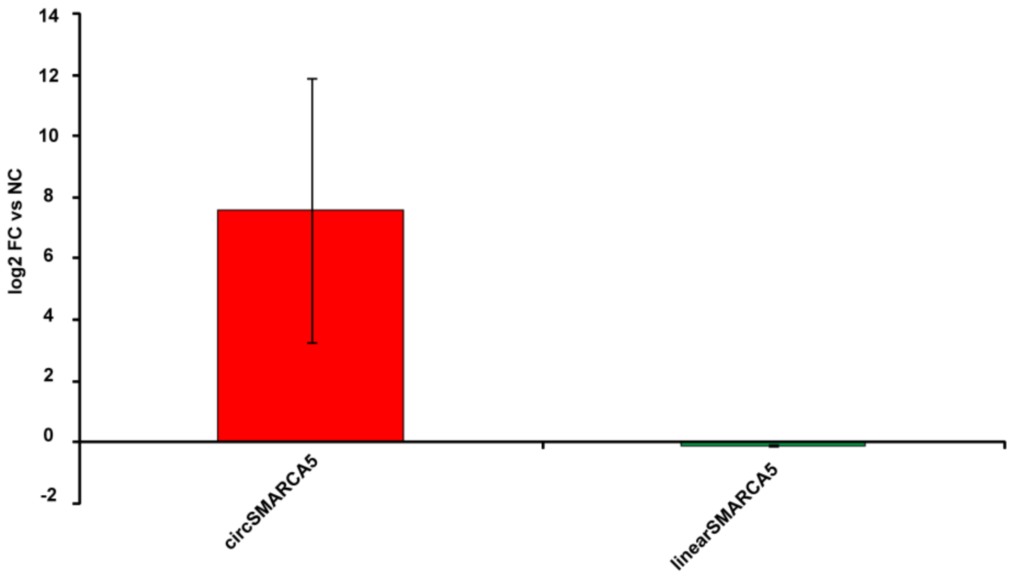

Figure S5. CircSMARCA5 but not linear SMARCA5 is overexpressed in U87-MG transfected with pcDNA3-circSMARCA5 vector with respect to NC (U87-MG transfected with the empty vector). Data are reported as log2 fold change (FC) *vs* NC.

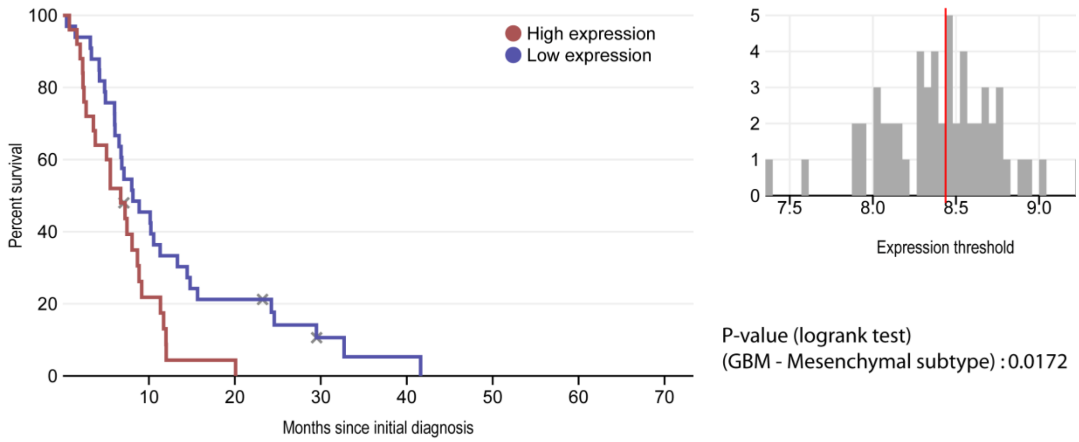

Figure S6. Kaplan-Meier overall survival curves of mesenchymal GBM patients, based on the expression of SRSF1. Patients having an higher expression of SRFS1 survive less than patients with a lower expression of SRSF1.

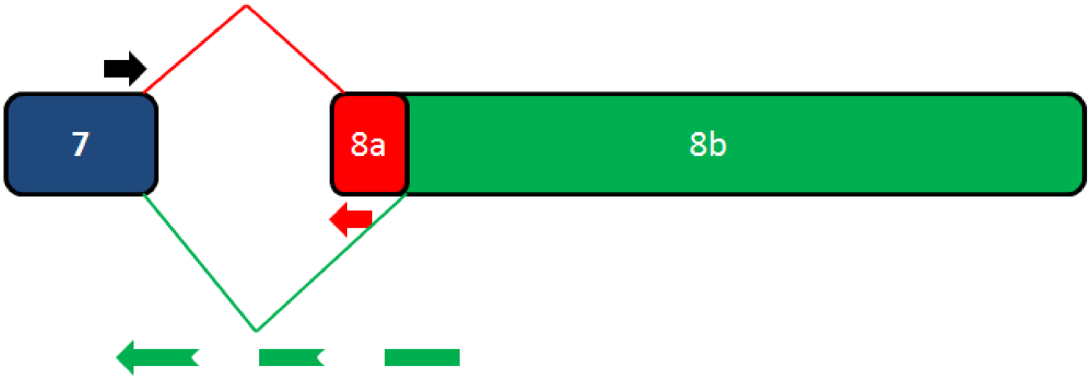

Figure S7. Diagram of primers used to amplify Iso8a and Iso8b VEGFA specific isoforms by qRT PCR. Forward primer, common to both isoforms is in black while Iso8a and Iso8b-specific reverse primers are in red and green, respectively.
